# Supplementary material for: Haloperidol Instigates Endometrial Carcinogenesis and Cancer Progression by the NF-κB/CSF-1 Signaling Cascade
Source: Cancers (Basel). 2022 Jun 23;14(13):3089. doi: 10.3390/cancers14133089 (PMC9265032; doi:10.3390/cancers14133089)
Supplement: Supplementary file 1 [file cancers-14-03089-s001.zip › cancers-1740937-supplementary.pdf]

Haloperidol instigates endometrial carcinogenesis and cancer progression by the NF-κB/CSF-1 signaling cascade

Supplementary Data

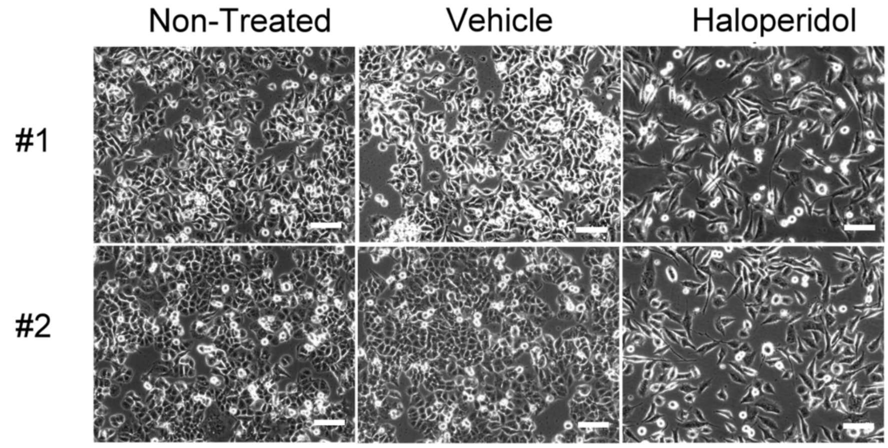

Figure S1. *The morphology characteristics of primary human endometrial cells derived from two patients (#1 and #2) treated with vehicle or haloperidol (100 μM) for 7 days.*

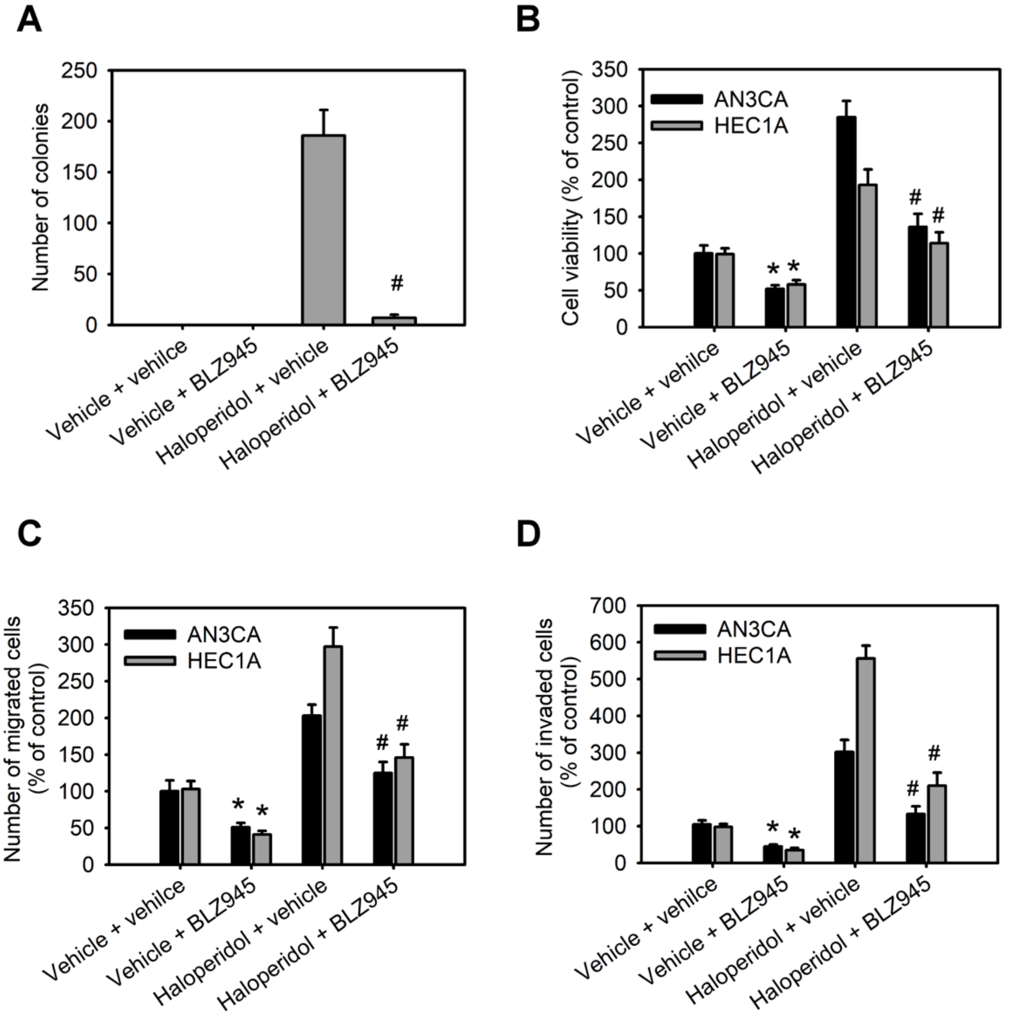

Figure S2. *Blockade of CSF-1R inhibits the cellular transformation of HEECs and the malignant progression of HECCs.* (**A**) The number of colonies formed in soft agar of HEECs treated with vehicle (normal saline) plus vehicle (20% Captisol®), vehicle (normal saline) plus BLZ945 (500 nM), haloperidol (100 µM) plus vehicle (20% Captisol®) and haloperidol (100 µM) plus BLZ945 (500 nM) for 28 days. Cell viability (**B**), migration (**C**), and invasion (**D**) of HECCs treated with vehicle (normal saline) plus vehicle (20% Captisol®), vehicle (normal saline) plus BLZ945 (500 nM), haloperidol (100 µM) plus vehicle (20% Captisol®) and haloperidol (100 µM) plus BLZ945 (500 nM) for 48 h. Data are presented as means ±SD within triplicate experiments. *p < 0.001, compared with the vehicle plus vehicle group. #p<0.001, compared with the haloperidol plus vehicle group.

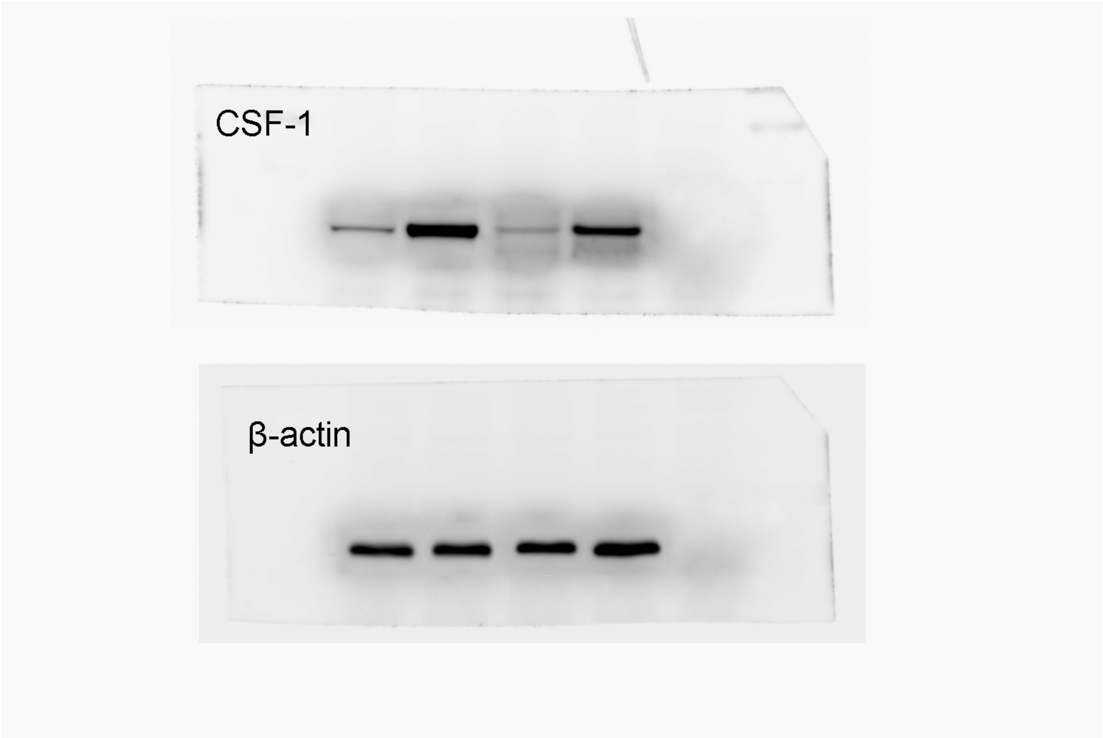

Figure S3. *Original uncut gel photo corresponding to Fig. 4C.*

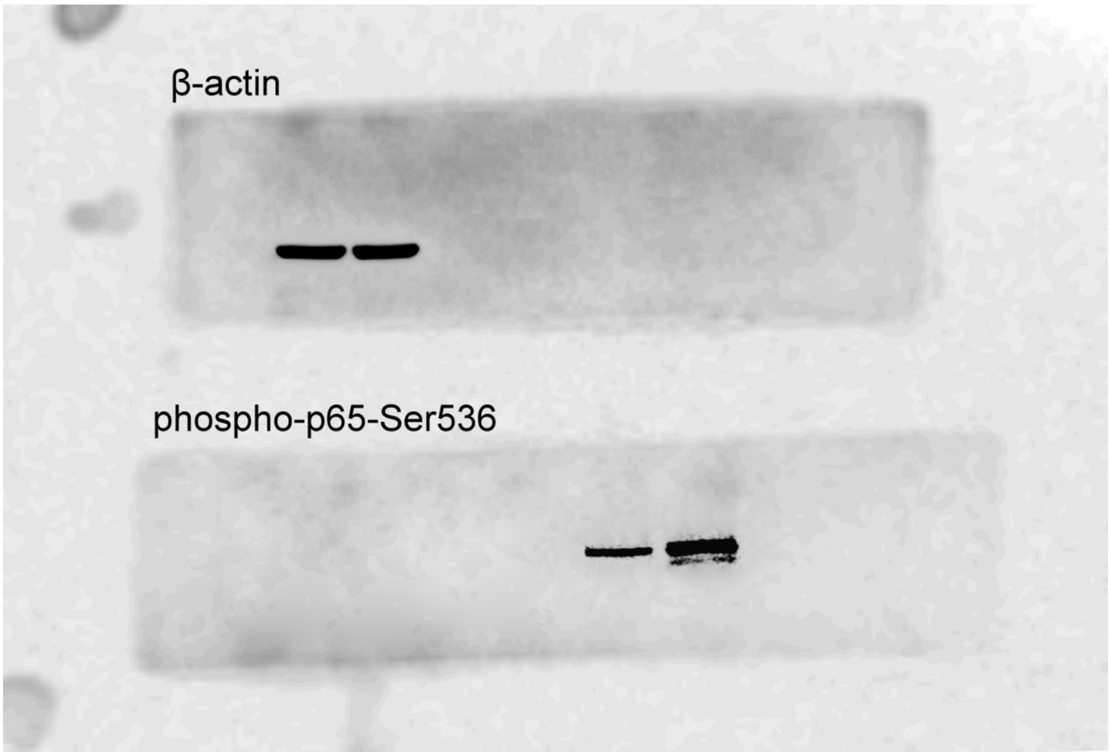

Figure S4. *Original uncut gel photo corresponding to Fig. 4G.*
